# The Molecular Landscape of the Senescence–EMT Interplay: Deciphering Tumour Plasticity Through Molecular Signatures and Multi-Omic Profiling

**DOI:** 10.3390/cancers18193204

**Published:** 2026-10-04

**Authors:** Eva M. Verdugo-Sivianes, Carmen Campos-Silva, José Luis García-Márquez, Elena Aguado-Domínguez

**Affiliations:** 1 Departamento de Biociencias, Facultad de Ciencias de la Salud, Universidad Loyola Andalucía, Avda. de las Universidades S/N, Dos Hermanas, 41704 Sevilla, Spain; ccampos@uloyola.es; 2 Facultad de Ciencias de la Salud, Universidad Loyola Andalucía, Avda. de las Universidades S/N, Dos Hermanas, 41704 Sevilla, Spain; jlgarciamarquez@al.uloyola.es; 3 Departamento de Ciencias Clínicas y de la Salud, Facultad de Ciencias de la Salud, Universidad Loyola Andalucía, Avda. de las Universidades S/N, Dos Hermanas, 41704 Sevilla, Spain; eaguado@uloyola.es

**Keywords:** cellular senescence, therapy-associated senescence (TIS), epithelial-to-mesenchymal transition (EMT), cellular plasticity, therapeutic resistance

## Abstract

Therapy-induced senescence (TIS) is a common response to anticancer treatments, characterised by stable cell cycle arrest and the acquisition of a complex senescence-associated secretory phenotype (SASP). Initially regarded as a beneficial mechanism that limits tumour growth, growing evidence indicates that TIS is a highly dynamic and heterogeneous process with context-dependent effects that can either suppress or promote tumour progression. The SASP is a central mediator of tumour plasticity, linking TIS to epithelial-to-mesenchymal transition (EMT), cancer stemness, microenvironmental remodelling, therapeutic resistance, and metastatic dissemination. These findings highlight the therapeutic potential of senotherapeutic approaches, including senolytic and senomorphic agents, as key components of one–two-punch therapeutic strategies to selectively eliminate or modulate persistent senescent cells and improve treatment outcomes. Recent advances in multi-omics technologies, artificial intelligence, and liquid biopsy are further transforming the study of senescence by enabling comprehensive molecular characterisation, biomarker discovery, patient stratification, and real-time monitoring of tumour evolution. Collectively, current evidence supports a paradigm shift in which senescence should be considered a dynamic regulator of tumour plasticity and a promising target for precision oncology strategies.

## Simple Summary

Cancer treatments can induce a stable cell cycle arrest in tumour cells, leading some of them to enter a state known as therapy-induced senescence. Senescent cells remain metabolically active and can release signalling molecules that affect surrounding cells. These signals can have different effects depending on the tumour and treatment context: they may contribute to tumour control, but they can also alter the tumour microenvironment and promote cellular changes associated with tumour progression and treatment resistance. This review examines how therapy-induced senescence may interact with processes such as epithelial-to-mesenchymal transition, tumour plasticity and cancer stemness. We also discuss emerging approaches for identifying and monitoring senescent cells, including multi-omics analyses, artificial intelligence and liquid biopsy. Understanding when senescence contributes to tumour control or, conversely, to tumour adaptation may help improve the development of more precise cancer treatments.

## 1. Introduction

Cellular senescence is a stress response mechanism characterised by a stable and generally long-lasting arrest of the cell cycle. It can be triggered by a variety of stimuli, including telomere shortening, DNA damage, oxidative stress, oncogene activation, and mitochondrial dysfunction [1,2,3,4]. Although senescent cells characteristically lose their proliferative capacity, they remain metabolically active and develop a characteristic secretory phenotype known as the senescence-associated secretory phenotype (SASP). This phenotype consists of a complex mixture of cytokines, chemokines, growth factors, proteases, and other bioactive molecules capable of altering the tissue microenvironment and mediating communication with neighbouring cells [2,3,4].

From a physiological perspective, cellular senescence plays essential roles in embryonic development, tissue repair, wound healing, and tumour suppression. By preventing genetically damaged cells from continuing to proliferate, it acts as one of the primary natural defence mechanisms of the organism against malignant transformation. However, when senescent cells persist and are not efficiently cleared by the immune system, sustained SASP secretion promotes chronic inflammation, disrupts tissue homeostasis, and contributes to ageing and the progression of several diseases, including cancer [1,3,5].

In this context, therapy-induced senescence (TIS) has emerged as a widely recognised response to anticancer treatment. Importantly, physiological senescence and TIS are not interchangeable, as they may differ substantially in their inducing stimuli, cellular context, molecular characteristics and functional consequences within the tumour microenvironment. Conventional therapies, including chemotherapy, radiotherapy, and several targeted therapies, can induce cellular stress and, depending on the treatment and cellular context, DNA damage sufficient to trigger senescence in both tumour cells and stromal cells within the tumour microenvironment [2,3]. Initially, this phenomenon was considered a desirable therapeutic outcome because durable cell cycle arrest effectively limited tumour cell proliferation, even when cancer cells were not eliminated through apoptosis [2,3]. However, increasing evidence over the last decade has demonstrated that TIS exhibits a markedly dual role. On the one hand, it exerts antitumour effects by durably suppressing malignant cell proliferation, enhancing immune surveillance, and promoting the immune-mediated clearance of damaged cells [2,3,6]. On the other hand, the long-term persistence of senescent cells can become detrimental. Importantly, the SASP is a temporally dynamic phenotype whose composition and functional effects can evolve during the establishment and persistence of senescence. Sustained or persistent SASP signalling can reshape the tumour microenvironment by promoting chronic inflammation, angiogenesis, epithelial-to-mesenchymal transition (EMT), immune evasion, cancer stemness, and stromal remodelling. Collectively, these alterations may contribute to therapy resistance, tumour recurrence, and metastatic progression [3,5,6].

Increasing evidence indicates that therapy-induced senescence should not be regarded as a uniform and invariably terminal endpoint, but rather as a heterogeneous and potentially dynamic response embedded within the broader framework of tumour cell plasticity. Phenotypic plasticity refers to the ability of genetically similar cells to adopt distinct functional states in response to environmental or microenvironmental cues, allowing tumours to adapt without the immediate acquisition of new genetic alterations. Through this process, cancer cells can reversibly transition among differentiated, stem-like, quiescent, invasive, and drug-tolerant states, and, in some contexts, dormant states, thereby supporting tumour initiation, progression, metastatic dissemination, and resistance to therapy [7]. Importantly, TIS may intersect with tumour dormancy, as anticancer treatment can contribute to the persistence or selection of residual tumour cell populations in prolonged non-proliferative states. However, dormancy encompasses a broader range of cellular states and should not be considered synonymous with senescence or the SASP [8,9]. From an evolutionary perspective, these transient phenotypic states may provide a rapid adaptive response to environmental challenges and create a window during which more stable genetic or epigenetic mechanisms of resistance can subsequently emerge [10]. In this context, it is also important to distinguish environmentally induced plasticity from phenotypic noise. Whereas therapeutic or microenvironmental pressures may actively induce adaptive cellular programmes, spontaneous non-genetic fluctuations can generate rare pre-existing subpopulations that are already capable of surviving subsequent therapeutic exposure [11].

Epithelial-to-mesenchymal plasticity, including epithelial-to-mesenchymal transition and its intermediate states, represents one of the best-characterised manifestations of tumour cell plasticity. Rather than constituting a binary switch between fully epithelial and fully mesenchymal phenotypes, EMT comprises a continuum of intermediate or hybrid states with different combinations of epithelial adhesion, mesenchymal motility, invasiveness, resistance to cellular stress, and stem-like properties. These reversible states allow cancer cells to adapt to changing microenvironmental conditions and to the selective pressures imposed by anticancer treatments. Similarly, the organisation of cancer stem cell populations is increasingly understood as a dynamic process rather than a rigid and unidirectional hierarchy. Experimental evidence has demonstrated that non-stem tumour cells can spontaneously acquire stem-like characteristics, indicating that the cancer stem cell compartment may be dynamically replenished during tumour evolution or following therapeutic depletion, and that eliminating pre-existing cancer stem cells alone may not be sufficient to prevent tumour recurrence [12]. These phenotypic transitions are also supported by metabolic plasticity, which enables cancer cells to adapt their use of glycolysis, oxidative phosphorylation, fatty acid oxidation, and glutamine metabolism according to microenvironmental conditions and therapeutic stress. In cancer stem cells, this metabolic flexibility can reinforce EMT, stemness, and treatment resistance through reciprocal interactions with transcriptional, epigenetic, and microenvironmental programmes. Metabolic reprogramming should therefore be considered both a consequence and a regulator of tumour cell plasticity [13].

Within this dynamic framework, therapy-induced senescence may influence tumour evolution through mechanisms that extend beyond the initial arrest of proliferation. Therapy-exposed tumour and stromal cells may differ considerably in the depth and stability of their cell cycle arrest, their metabolic configuration, the composition of their SASP, and their interactions with the immune system. This heterogeneity may ultimately determine whether TIS promotes immune-mediated tumour clearance or instead generates a microenvironment that facilitates the survival and adaptation of residual cancer cells [14]. Importantly, this context dependence may limit the generalizability of senescence-associated findings across tumour types and patient populations, highlighting the need for tumour- and context-specific validation. In particular, SASP-mediated paracrine signalling may expand the phenotypic repertoire of neighbouring cells by promoting EMT, stem-like properties, inflammatory signalling, and resistance-associated programmes [15,16]. At the same time, therapeutic pressure may either induce these adaptive phenotypes or select pre-existing cellular states that are more capable of tolerating treatment [7,11]. Senescence, EMT, and cancer stemness should therefore not be considered interchangeable phenomena. Instead, a central challenge is to determine when these states coexist, when they causally reinforce each other, and when they represent independent or divergent cellular trajectories [13,14].

This paradigm shift has driven the development of novel therapeutic strategies aimed at preserving the beneficial effects of TIS while minimising its adverse consequences [17]. These approaches include senolytic agents, which selectively eliminate senescent cells, and senomorphic drugs, which suppress or modulate detrimental SASP components without necessarily inducing cell death, although their effects may be context-dependent and may also influence immune-mediated surveillance of senescence cells [17,18]. Nevertheless, their successful clinical implementation requires the identification of the specific senescent populations that should be targeted, as well as the optimal therapeutic window in which their elimination or modulation would provide the greatest benefit [17]. This remains particularly challenging because senescence is not defined by a single universal marker and its molecular features vary according to cell type, tumour context, senescence-inducing stimulus, and time elapsed after treatment [19,20]. Commonly used senescence-associated markers, including SA-β-galactosidase activity, p16^INK4a^, p21^CIP1^ and components of the SASP, can support the identification of senescence but lack absolute specificity, while their absence does not necessarily exclude it. This is particularly relevant in tumours, where senescent cells are heterogeneous and may coexist with quiescent, stressed, differentiated or other cell states. Therefore, a combination of complementary markers and functional information is likely to provide a more robust assessment, although the lack of a universally specific and clinically validated marker remains an important barrier to clinical translation.

Emerging technologies offer new opportunities to resolve this complexity. Single-cell and spatially resolved multi-omics approaches can characterise coexisting senescent, epithelial, mesenchymal, stem-like, and immune cell states while preserving information about their molecular features and spatial organisation [19,21]. Longitudinal sampling and liquid biopsy approaches may further support the minimally invasive monitoring of therapy-associated molecular changes and the emergence of resistant cellular populations [22]. In parallel, machine learning and artificial intelligence-based methods can integrate high-dimensional transcriptomic, epigenomic, proteomic, metabolomic, and clinical data to identify composite senescence-associated signatures, infer cellular trajectories, stratify patients, and uncover previously unrecognised therapeutic vulnerabilities [19,23]. However, these computational models require robust biological annotation, longitudinal validation, and independent clinical cohorts to distinguish clinically meaningful plasticity from transient or non-specific stress responses [23].

Despite these advances, the available evidence remains heterogeneous and, in some cases, contradictory regarding the overall impact of TIS on tumour progression and therapeutic response [24]. This apparent duality raises a central question: which factors determine whether TIS contributes to tumour suppression or, alternatively, to tumour adaptation and progression? TIS can be considered a dynamic, context-dependent state whose effects are shaped by interactions between senescent cells, SASP signalling, tumour cell plasticity, EMT, cancer stemness and the tumour microenvironment. These interconnected processes may influence tumour cell adaptation to therapeutic pressure and contribute to therapeutic resistance or tumour progression in specific contexts, rather than following a universal or unidirectional trajectory.

Accordingly, this review examines the interplay between TIS, EMT, cellular plasticity, cancer stemness, and tumour progression and considers how emerging single-cell and multi-omics technologies, artificial intelligence, machine learning, and liquid biopsy approaches may help characterise these heterogeneous states and identify predictive biomarkers and actionable molecular vulnerabilities to support the development of more effective and personalised therapeutic strategies [17,19,20,21].

To identify relevant literature, we conducted a narrative search of PubMed/MEDLINE using combinations of terms related to therapy-induced senescence, cellular senescence, the senescence-associated secretory phenotype, epithelial-to-mesenchymal transition, tumour plasticity, therapeutic resistance, tumour evolution, multi-omics, single-cell analysis, artificial intelligence, machine learning and liquid biopsy, together with cancer-related terms. The search covered the relevant literature published up to July 2026, and additional studies were identified through reference screening of relevant articles and reviews. Studies were selected based on their relevance and biological, methodological, or clinical contribution, with consideration of the level of supporting evidence.

## 2. Senescence as a Driver of EMT and Cellular Plasticity

EMT is a key mechanism of cellular plasticity that allows epithelial cells to change their phenotype in response to environmental signals. During this process, cells lose their cell–cell adhesion and polarised epithelial characteristics and acquire a more invasive mesenchymal phenotype. This transition facilitates the dissemination of cancer cells from the primary tumour and contributes to metastatic colonisation of distant organs. EMT is commonly associated with downregulation of epithelial markers, particularly E-cadherin, and the increased expression of mesenchymal proteins such as vimentin and N-cadherin, although these changes are not necessarily reciprocal or complete in partial or hybrid EMT states. These molecular changes are driven by EMT-associated transcription factors, including Snail family transcriptional repressor 1 and 2 (*SNAI1* and *SNAI2/SLUG*), Twist family BHLH transcription factor 1 (*TWIST1*), and zinc finger E-box-binding homeobox 1 and 2 (*ZEB1/2*), which repress epithelial genes while promoting mesenchymal gene expression [25,26,27]. Beyond promoting migration and metastasis, EMT is closely associated with the acquisition of cancer stem cell (CSC) properties, including enhanced self-renewal capacity, tumour-initiating potential, and resistance to apoptosis and anticancer therapies. The SASP can contribute to this transition in certain contexts through the release of inflammatory factors, including interleukins (IL-6, IL-8), and transforming growth factor-β (TGF-β), which have been implicated in the induction of EMT-associated phenotypes in neighbouring cells. Conversely, EMT-associated cells can modify the inflammatory tumour microenvironment by producing additional cytokines and signalling molecules, potentially creating a positive feedback loop that supports CSC maintenance, tumour progression and therapeutic resistance [25,28,29,30].

**Figure 1:**
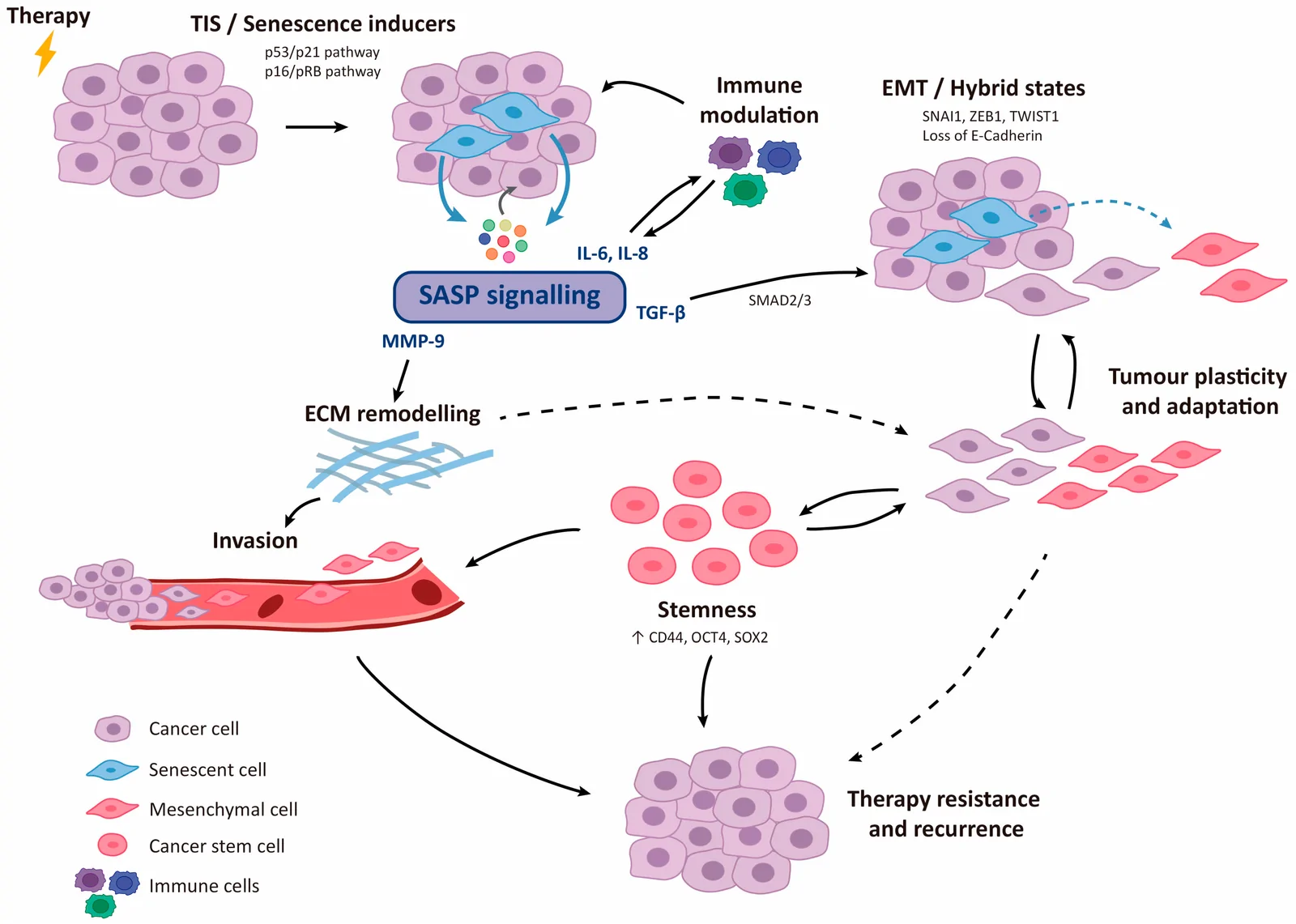
Mechanistic interplay between therapy-induced senescence (TIS) and EMT-driven metastasis. Exposure to genotoxic treatments can induce a state of persistent growth arrest known as therapy-induced senescence (TIS), characterised by the activation of p53/p21 and p16/Rb pathways. These senescent cells can develop a senescence-associated secretory phenotype (SASP) rich in pro-inflammatory cytokines (e.g., IL-6, IL-8), growth factors (e.g., TGF-β) and matrix metalloproteases (MMP-9). Through paracrine signalling, the SASP triggers the epithelial-to-mesenchymal transition (EMT) in neighbouring cancer cells via transcription factors like SNAI1 and ZEB1. This phenotypic remodelling facilitates the acquisition of cancer stem cell (CSC) properties and high tumour plasticity, ultimately bypassing senescence-induced growth arrest to promote tumour dissemination and metastatic progression. Solid arrows indicate mechanisms or relationships supported by established evidence, whereas dashed arrows indicate proposed, less well-established, or predicted relationships.

Senescence and EMT have traditionally been viewed as distinct, and in some cases opposing, cellular programmes. However, recent evidence indicates that these processes can intersect through shared signalling pathways due to a cellular adaptation to stress, although the nature and direction of this relationship appear to be highly context-dependent. In particular, senescent cells may influence EMT-associated phenotypes in neighbouring cells through paracrine signalling, whereas EMT can also arise independently of senescence or precede the acquisition of senescence-associated features. Thus, the available evidence supports functional crosstalk between senescence and EMT in specific cellular and tumour contexts, rather than a universal relationship between the two states (Figure 1) [16].

Overexpression of *ZEB1* in cancer promotes EMT and increases the metastatic potential. Conversely, the loss of function or mutation of *ZEB1* in murine models can promote the reverse process, a mesenchymal-to-epithelial transition (MET), characterised by increased epithelial features, including E-cadherin expression, together with reduced mesenchymal features, including decreased vimentin expression in this experimental context. This reprogramming of cellular identity is associated with diminished proliferation in progenitor cells and premature senescence in mouse embryonic fibroblasts. Crucially, this replicative arrest occurs independently of the classic p16 (*CDKN2A*) pathway. Instead, ZEB1 acts as a direct transcriptional repressor of the cyclin-dependent kinase inhibitors p15 (*CDKN2B*) and p21 (*CDKN1A*). These findings illustrate that EMT-associated transcriptional regulators can also influence cell cycle control and senescence pathways, providing a potential molecular point of convergence between EMT and senescence [31].

TGF-β signalling represents another important molecular link between senescence-associated signalling and EMT and has a well-established dual and context-dependent role in cancer. In normal tissues and during the early stages of carcinogenesis, TGF-β maintains tissue homeostasis by inducing cell cycle arrest through the SMAD (suppressor of mothers against decapentaplegic)-dependent activation of cyclin-dependent kinase inhibitors, including p15, p16, and p21. In addition, it can promote a SASP that may contribute to immune-mediated clearance of damaged cells while preserving extracellular matrix integrity. However, the effects of TGF-β during tumour progression are highly dependent on cellular, molecular and microenvironmental context. In established tumours, alterations in TGF-β signalling and the balance between Smad-dependent and Smad-independent pathways can favour oncogenic processes, including EMT, invasion and metastasis, through the loss of E-cadherin and acquisition of mesenchymal traits, as well as immunosuppressive tumour microenvironment remodelling. Furthermore, TGF-β has been implicated in cancer stem cell maintenance, angiogenesis, and therapeutic resistance. These context-dependent functions have important therapeutic implications. Although inhibition of TGF-β signalling may be beneficial in advanced tumours, where its pro-tumour and immunosuppressive effects may predominate, its blockade in earlier stages could interfere with its tumour-suppressive functions. Thus, the therapeutic exploitation of TGF-β may require context- and stage-specific modulation, considering tumour type, disease stage and biological context [32]. Therapy-induced senescence not only limits tumour proliferation, but also it is a potent modulator of the tumour microenvironment by a paracrine promotion of malignant progression. Upon exposure to genotoxic agents such as 5-fluorouracil (5-FU) or doxorubicin, colon cancer cells enter in senescence and produce a senescent-associated secretome (SAS) enriched in IL-8 and TGF-α. This SAS triggers the EMT in adjacent non-senescent cells by inducing the loss of E-cadherin and the increase in SLUG, ZEB1, SNAI1, fibronectin and vimentin. These molecular alterations significantly enhance the invasive capacity and aggressive behaviour of tumour cells [33].

Interestingly, although senescent cells initiate the process, they express relatively low levels of EMT markers compared with the neighbouring cells exposed to their secretome. This observation supports a paracrine effect in this experimental setting. Consistent with these findings, analyses of rectal cancer samples from patients undergoing neoadjuvant chemoradiotherapy identified tumour regions enriched in senescent cells adjacent to areas with increased expression of EMT markers. Although these observations support a potential association between TIS and EMT in this clinical context, they do not themselves establish a causal relationship in human tumours [33].

The implications of EMT are particularly significant in ovarian cancer, given its unique pattern of metastatic dissemination. Ovarian cancer cell dissemination primarily occurs as cells detach from the primary tumour and spread into the peritoneal cavity. During this process, EMT promotes the loss of epithelial characteristics and the acquisition of migratory and invasive properties, facilitating metastatic colonisation. Those cells must overcome anoikis, a form of programmed cell death triggered by the loss of cell–matrix adhesion. This progression is heavily supported by the tumour microenvironment, which provides a permissive niche rich in inflammatory cytokines, chemokines and metalloproteinases [34].

The role of the tumour microenvironment in ovarian cancer is further supported by the contribution of senescent fibroblasts, which accumulate at primary tumour sites and within the deeper layers of the peritoneum. Beyond ovarian cancer, these cells are known to facilitate dissemination in pancreatic and breast cancers; they facilitate dissemination by shaping an immunosuppressive microenvironment and inducing EMT [35]. In the specific context of ovarian cancer, previous works have reported that senescent fibroblasts promote the malignant transformation of ovarian epithelial cells while enhancing their proliferation, migration and invasion [36,37].

A defining feature of fibroblasts is their capacity to drive extracellular matrix (ECM) remodelling. Recent profiling of senescent ovarian fibroblasts showed that their SASP is composed of different inflammatory and immune-modulatory cytokines, chemokines and ECM remodelling regulators. This secretome, while not enhancing cancer cell proliferation, enhances the migratory capacity and confers resistance to apoptosis, potentially contributing to metastasis formation and cancer dissemination. In this study, mapping of the secretome revealed the upregulation of senescent-associated factors such as the receptor for advanced glycation end products (RAGE), IL-6 and the growth differentiation factor 15 (GDF-15), which have previously been involved in EMT and cancer cell migration in ovarian and other types of cancer. Novel cytokines such as chitinase-3-like protein 1 (CHI3L1) and fibroblast growth factor 7 (FGF-7), which are not yet established as senescence markers, but whose overexpression has been associated with chemoresistance and ovarian cancer metastasis, were also identified [34]. A pivotal finding of this study is the first report of the co-secretion of matrix metalloproteinase-9 (MMP-9) and neutrophil gelatinase-associated lipocalin (NGAL) by senescent ovarian fibroblasts. NGAL binds and stabilises MMP-9, a key enzyme involved in ECM degradation and associated with ageing and tumour invasion. In the experimental model investigated, the axis NGAL-MMP-9 triggers a cadherin switch—downregulation of E-cadherin and upregulation of vimentin, thereby driving the EMT-like phenotype and anoikis resistance necessary for systemic dissemination [34].

Beyond neoplastic contexts, the coordination between cellular identity and growth arrest is further exemplified by the transcription factor GATA-binding protein 3 (GATA3) during placental development. Recent evidence demonstrates that the suppression of GATA3 in extravillous trophoblasts simultaneously triggers EMT and cellular senescence. At the molecular level, GATA3 deficiency promotes a phenotypic shift characterised by the downregulation of epithelial markers, such as E-cadherin, and the induction of mesenchymal factors, including vimentin and matrix metalloproteinase-2 (MMP-2). This reprogramming is coupled with an accelerated cellular senescence, evidenced by increased senescence-associated β-galactosidase activity and the upregulation of key senescence effectors, such as p21, p16 and p53. The loss of GATA3 also leads to the activation of EMT-inducing transcription factors, specifically ZEB1/2, TWIST1 and SNAI2 [38].

In summary, current evidence supports a context-dependent interplay between cellular senescence and EMT that may contribute to tumour plasticity, promoting cancer progression, metastasis, and therapeutic adaptation in specific settings. Rather than representing interchangeable or obligatory linked cellular states, senescence and EMT may coexist, influence one another indirectly through paracrine signalling or appear as distinct cellular responses to therapeutic environmental stress. Defining the circumstances under which these states are causally connected, rather than merely correlated, will be important for identifying clinically relevant biomarkers and novel therapeutic vulnerabilities.

## 3. Therapy-Induced Tumour Evolution and Adaptation

Therapy-induced tumour evolution is shaped by molecular and microenvironmental differences between primary tumours and metastatic lesions, potentially contributing to heterogeneous responses to anticancer therapy [3]. Comparative genomic studies have demonstrated that metastatic lesions may acquire distinct molecular alterations during tumour progression and under therapeutic pressure, although the extent of this divergence varies across cancer types [39,40]. Recent single-cell analyses of non-small-cell lung cancer have identified differences in senescence-associated states between primary tumours and metastatic sites, suggesting that the local tumour context may influence the induction and consequences of TIS [3]. Thus, site-specific tumour evolution may represent an additional source of heterogeneity in TIS and may contribute to differences in subsequent therapeutic adaptation and resistance [3].

### 3.1. Chemotherapeutic Resistance

The emergence of senescent cells following chemotherapy may contribute to a permissive environment that fosters cellular plasticity and metastasis. Importantly, tumour dormancy may also contribute to the persistence of residual tumour cells following treatment [8,9]. Senescence-associated signalling and EMT represent only part of a broader and multifactorial network of resistance mechanisms, which can also involve genetic and epigenetic alterations, changes in drug targets and transport, altered drug metabolism, and evasion of cell death, among others. Consequently, the combined assessment of senescence and EMT biomarkers is proposed as a promising strategy for predicting tumour aggressiveness and guiding therapeutic decisions in the post-chemotherapy setting.

The molecular mechanisms underlying metabolic escape from TIS may be driven, at least in part, by the SIRT5-MTHFD2 axis in breast cancer. Sirtuin 5 (SIRT5), a mitochondrial NAD^+^-dependent desuccinylase, regulates redox homeostasis by modulating methylenetetrahydrofolate dehydrogenase 2 (MTHFD2), a key enzyme in mitochondrial one-carbon metabolism that contributes to NADPH production. While SIRT5 has been linked to cellular senescence, antioxidant defence and tumour progression, MTHFD2 is frequently overexpressed in breast cancer, where it sustains metabolic adaptation and proliferation. Conversely, reduced MTHFD2 expression or activity promotes metabolic stress, mitochondrial dysfunction and cellular senescence, highlighting its potential as a therapeutic target [41].

Tumour cells rely on NADH-dependent antioxidant defence mechanisms to prevent reactive species oxygen (ROS) from reaching levels that trigger senescence or apoptosis. Indeed, overexpression of NADPH-generating enzymes is a common metabolic adaptation that preserves redox homeostasis and supports tumour survival. Recent evidence has identified MTHFD2 succinylation as a key regulator of this process. Succinylation of MTHFD2 at K44 inhibits its enzymatic activity, reducing NADPH production, increasing ROS accumulation and activating the AMP-activated protein kinase (AMPK)/p53/p21 signalling pathway, thereby promoting senescence and enhancing chemosensitivity. In contrast, SIRT5-mediated desuccinylation restores MTHFD2 activity, maintains antioxidant capacity and prevents ROS accumulation from reaching the threshold required for TIS, ultimately promoting resistance to agents such as olaparib.

Since many chemotherapeutic agents exert their antitumour effects by inducing DNA damage and oxidative stress, activation of the SIRT5–MTHFD2 axis enables tumour cells to escape TIS and survive treatment. Consistently, breast cancer cells with SIRT5 knockdown exhibit increased MTHFD2 succinylation and enhanced sensitivity to chemotherapy in vivo. These findings identify the SIRT5-MTHFD2 axis as a promising metabolic vulnerability and suggest that targeting MTHFD2 or succinylation-regulating enzymes, particularly in combination with senolytic therapies, may enhance chemotherapy efficacy while limiting the detrimental effects associated with persistent senescent cells [41].

Given the dual role of senescence in cancer, therapeutic strategies should not only promote tumour growth arrest but also prevent the long-term detrimental effects associated with persistent senescent cells. In this context, natural compounds such as verbascoside have emerged as promising modulators of senescence-associated cellular plasticity.

Verbascoside, a phenylethanoid glycoside, has shown anti-senescence and anti-metastatic effects in preclinical models of castration-resistant prostate cancer (CRPC), where it appears to modulate mitochondrial homeostasis, invasive behaviour and cell cycle regulation. Verbascoside suppresses EMT by restoring *E-cadherin* expression and downregulating mesenchymal genes such as *SNAI1* and *SNAI2/SLUG*, thereby reducing metastatic potential. In addition, it acts as a senomorphic agent by suppressing the SASP and reducing the secretion of pro-inflammatory cytokines, including IL-6, IL-8, and IL-1. Mechanistically, verbascoside attenuates pro-senescence signalling through the downregulation of p38 mitogen-activated protein kinase (p38 MAPK), p16, p21 and p27, together with the upregulation of retinoblastoma protein (pRB) levels. Furthermore, verbascoside enhances antioxidant defence by increasing superoxide dismutase (SOD) activity, which reduces oxidative stress damage and slows cancer progression. In addition, it stimulates mitochondrial biogenesis through the AMPK/peroxisome proliferator-activated receptor gamma coactivator 1-alpha (PGC-1α)/nuclear respiratory factor 1 (NRF1) axis, a process crucial for maintaining energy homeostasis, counteracting the deleterious effects of oxidative stress and cellular senescence. Consequently, these findings suggest that verbascoside modulates senescence, EMT and mitochondrial-related pathways in preclinical CRPC models. However, its clinical potential remains uncertain due to limited bioavailability and the lack of clinical evidence [42].

These strategies exploit mitochondrial metabolic and oxidative pathways to enable tumour cells to evade or overcome TIS. However, this process is also orchestrated at the epigenetic level. Regulators such as Chromobox homolog 7 (CBX7) are critical to repress senescence and maintain malignant proliferation and chemoresistance. CBX7 is a subunit of the Polycomb Repressive Complex 1 (PRC1), a multiprotein complex that participates in the epigenetic regulation of gene expression, which regulates cell proliferation, migration and senescence. CBX7 exhibits dual role in tumorigenesis, in some tumours acts as a tumour suppressor but in others acts as an oncogene. In the context of multiple myeloma, clinical data showed that CBX7 was highly expressed in myeloma, suggesting a tumour-promoting role [43].

Bortezomib (BTZ)-based chemotherapy effectively induces cellular senescence in multiple myeloma. CBX7 expression was reduced during BTZ-induced senescence in myeloma cells, and its knockdown further promoted senescence both in vitro and in vivo, suggesting that CBX7 acts as a suppressor of the senescence programme. Therefore, targeting CBX7 may enhance the efficacy of standard chemotherapy for multiple myeloma. At the mechanistic level, CBX7 modulates cellular senescence through the regulation of the extracellular signal-regulated kinase (ERK)/signal transducer and activator of transcription 3 (STAT3)/proviral integration site for Moloney murine leukaemia virus 1 (PIM1) signalling axis. The kinase PIM1, a critical downstream effector negatively regulated by CBX7, is upregulated during BTZ-induced senescence and contributes to the establishment of the senescent phenotype, at least in part through activation of the p53/p21 pathway and induction of p16 expression, thereby reinforcing cell cycle arrest. Since the MAPK-ERK pathway was overactivated during BTZ-induced senescence and negatively regulated by CBX7, the treatment with ERK inhibitors seems to attenuate CBX7-mediated senescence and enhance the inhibitory effects of bortezomib on myeloma cells. Taken together, these findings highlight the ERK/STAT3/PIM1 axis as a critical mediator of CBX7-dependent regulation of senescence and support CBX7 inhibition as an innovative therapeutic strategy to enhance bortezomib efficacy, promote senescence, and overcome chemoresistance in multiple myeloma [43]. Malignant cells can use the state of senescence to persist after treatment while simultaneously acquiring unique metabolic and molecular vulnerabilities. Senescent glioma cells undergo a significant metabolic switch towards glycolysis after treatment with temozolomide (TMZ). This state is characterised by the upregulation of death receptor 5 (DR5), one of the five receptors of the cytokine tumour necrosis factor-related apoptosis-inducing ligand (TRAIL), which triggers apoptosis upon ligand binding. As a component of immune surveillance, the TRAIL signalling pathway contributes to the elimination of transformed cells. DR5 upregulation after TMZ treatment sensitises senescent glioma cells to DR5-targeted TRAIL variants (DR5-B) due to activation of the TRAIL signalling pathway, providing a potential senolytic strategy for their selective elimination, although this sensitivity is highly dependent on the tumour’s genetic status, being most effective in p53-proficient and methylated-DNA–protein-cysteine methyltransferase (MGMT)-deficient contexts [44].

Beyond metabolic and epigenetic control, the SASP itself acts as a dynamic driver of therapy resistance. BRCA1-mutated triple-negative breast tumours show sensitivity to cyclin-dependent kinase 4 and 6 (CDK4/6) inhibitors such as abemaciclib, but this treatment triggers senescence and the secretion of a SASP rich in IL-6. The elevated IL-6 secretion leads to the aberrant activation of the STAT3 signalling pathway, which acts as a critical bypass mechanism to promote survival, migration and invasion despite cell cycle blockade. To overcome this resistance, the co-administration of bazedoxifene, a selective estrogenic receptor modulator and inhibitor of the IL6/STAT3 signalling pathway, has shown a strong synergistic effect when combined with abemaciclib. Therefore, targeting the IL6/STAT3 pathway may enhance the antitumour efficacy of CDK4/6 inhibition. In vivo studies further confirm that this combination markedly suppresses tumour growth without notable toxicity, offering a promising “one–two-punch” approach to sensitise resistant BRCA1-mutant triple-negative breast cancer and prevent systemic dissemination [45].

Collectively, the evasion of therapy-induced senescence is a multi-layered process involving very different pathways. The relative contribution of these mechanisms may vary according to tumour type, chemotherapeutic agent and cellular microenvironmental context, and their comparative importance across cancer types remains to be established. Understanding these distinct yet complementary mechanisms of escape is fundamental for developing next-generation treatments that can effectively sensitise resistant tumours to standard chemotherapy.

### 3.2. Radiotherapy-Induced Senescence

Radiotherapy induces DNA damage and oxidative stress, which can trigger therapy-induced senescence in tumour cells and components of the tumour microenvironment. Depending on dose, cellular context and the persistence of the senescent state, this response can initially constrain tumour proliferation but may also result in the accumulation of persistent senescent cells and SASP-mediated remodelling of the tumour microenvironment [46,47,48].

Different experimental studies indicate that radiotherapy-induced senescence can contribute to radioresistance. In head-and-neck squamous cell carcinoma, early radiation-induced senescence was positively associated with radioresistance and accompanied by NF-κB-dependent production of senescence-associated cytokines, while pharmacological inhibition of this secretory programme enhanced the response to radiotherapy [49]. Similar mechanisms have been described in glioblastoma, where radiation-induced senescent cells can promote proliferation and radioresistance of neighbouring tumour cells through SASP signalling, contributing to tumour recurrence [50]. These findings suggest that the persistence of radiation-induced senescent cells may represent an additional mechanism of tumour adaptation following radiotherapy and provide a rationale for combining radiotherapy with strategies targeting senescent cells or their deleterious secretory programmes.

Beyond these effects on tumour cell behaviour, radiotherapy-induced senescence may also reshape antitumour immunity. Recent single-cell analyses of prostate cancer have shown that radiation-resistant tumour cells can acquire senescence-associated states that influence their response to immune checkpoint blockade [51]. Thus, the immunomodulatory consequences of TIS may represent an additional mechanism through which radiotherapy shapes tumour adaptation and therapeutic response, providing a direct link between radiotherapy-induced senescence and the modulation of the immune system response.

### 3.3. Modulation of the Immune Response

The interplay between cellular senescence and the immune system is highly complex, as the persistent accumulation of senescent cells and their SASP can promote a pro-tumorigenic and immunosuppressive microenvironment. In bladder urothelial carcinoma (BLCA), cellular senescence has been closely linked to immune evasion and the response to immunotherapy. Among senescence-related genes, the progestin and adipoQ receptor family member 4 (*PAQR4*) gene has emerged as a key biomarker linked to poor prognosis, an immunosuppressive tumour microenvironment and immune escape in BLCA. Persistent senescent cells in BLCA secrete SASP factors that may initially recruit immune cells for their clearance; however, they can also establish a microenvironment that supports tumour progression, invasion and metastasis of neighbouring non-senescent cells. Consistent with this pro-tumourigenic context, PAQR4 knockdown suppresses BLCA cell proliferation and invasiveness, highlighting its potential as a novel therapeutic target to overcome immune evasion and limit tumour dissemination [52].

Lung adenocarcinoma (LUAD) is generally characterised by a high degree of immune cell infiltration, making it an attractive candidate for immunotherapy. Nevertheless, only a subset of patients with advanced non-small-cell lung cancer (NSCLC) sustain benefit from immune checkpoint inhibitor monotherapy, and the mechanisms underlying this resistance remain poorly understood [53]. CDKN1A, which encodes the cyclin-dependent kinase inhibitor p21, has been regarded as a tumour suppressor that inhibits cell proliferation in response to cellular stress. However, accumulating evidence indicates that p21 also exerts pro-tumourigenic functions by promoting cell migration, invasion, metastasis, and resistance to anticancer therapies. Indeed, elevated p21 expression has been associated with resistance to 5-FU in colorectal cancer and cisplatin in testicular and ovarian cancers, highlighting its potential as a predictive biomarker of chemotherapy response. Until recently, however, its role in immunotherapy resistance had remained largely unexplored [53].

Recent studies have demonstrated that elevated *CDKN1A* expression is associated with a senescence-associated “cold” tumour-immune phenotype in advanced LUAD, characterised by reduced immune cell infiltration and impaired immune recognition of tumour cells. This phenotype correlated with decreased infiltration of CD8^+^ and memory CD4^+^ T cells, together with activation of the TGF-β/SMAD signalling pathway, which promotes EMT and tumour stromal remodelling. Moreover, several senescence-related genes, including *INHA*, *TNFRSF10C*, *IGFBP2*, and *CXCL1*, have also been associated with poor responses to immune checkpoint blockade. Collectively, these findings suggest that p21-driven senescence may contribute to immune evasion and immunotherapy resistance in LUAD, highlighting senescence-associated pathways as promising therapeutic targets and potential biomarkers for improving patient stratification in precision oncology [53].

In contrast to the pro-tumourigenic and immune-evasive role associated with the senescence-related genes *PAQR4* in BLCA and *CDKN1A* in LUAD, breast cancer cells exhibit a senescent-like dormant phenotype to survive within the metastatic niche. These cells show an EMT phenotype to facilitate their dissemination and re-adhesion at secondary sites. This phenotype is characterised by downregulated nuclear-encoded oxidative phosphorylation (OXPHOS) transcripts, although they preserve ATP production, and upregulation of stemness markers. Notably, these cells present reduced programmed death-ligand 1 (PD-L1) expression, rendering senescent-like metastatic cells more susceptible to immune recognition and elimination [54]. Conversely, therapy-induced senescent cancer cells have been shown to upregulate PD-L1, contributing to immune evasion and persistence through reduced susceptibility to T-cell-mediated killing [55]. This finding suggests that senescence does not uniformly promote immune escape and may instead generate exploitable immunological vulnerabilities depending on the cellular and tumour context [54,55].

Consequently, the impact of senescence on antitumour immunity appears to be highly context- and tumour-specific, highlighting the need for personalised therapeutic strategies that consider the distinct immune landscapes associated with different senescent states.

## 4. Characterisation of Senescent Subpopulations and Clinical Stratification Using a Multi-Omics Strategy

The clinical management of cancer is increasingly relying on precision medicine to overcome the inherent heterogeneity of cancer and, more specifically, of cellular senescence. Traditional markers often fail to provide a universal definition of the senescent state across different tissues and therapeutic contexts. To address this challenge, current research integrates single-cell multi-omics and machine learning approaches to identify patterns and candidate gene signatures associated with senescence across different tumour types and biological contexts (Table 1).

A single-cell study was performed in nasopharyngeal carcinoma to decode cellular heterogeneity of senescent cells, identifying two key senescent subpopulations associated with malignancy: the epithelial C3 cluster and SPP1+ (secreted phosphoprotein/osteopontin-positive) macrophages. The senescent C3 cells drive EMT and metastasis, while conferring angiogenic capacity to the niche through paracrine signalling of SASP factors. These senescent cells present a metabolic reprogramming, characterised by high glycolytic activity and the production of lactic acid, which enhances drug resistance and tumour malignancy. Furthermore, a novel communication axis has been identified where senescent epithelial cells interact with SPP1+ macrophages via angiopoietin-like 4 (ANGPTL4). This axis determines the functional fate of macrophages by regulating the balance between glucose and lipid metabolism through receptors like syndecan-2 (SDC2). Consequently, the targeted removal of these senescent cells or the inhibition of the ANGPTL4/SDC2 axis represents a promising therapeutic strategy to disrupt the chronic inflammatory and immunosuppressive environment that supports cancer progression [56].

The potential of multi-omics integration is further illustrated in colorectal liver metastasis by the identification of a five-gene epigenetic–transcriptomic signature (CXCL1, SERPINE1, NDRG1, SRM, and GATM). By integrating genome-wide DNA methylation profiles with bulk RNA-sequencing data and curated gene sets related to cellular senescence and metabolic reprogramming, this machine learning-derived signature was shown to functionally couple senescence-associated secretory traits with metabolic plasticity, providing a robust prognostic biomarker in colorectal liver metastasis while also serving as a predictive biomarker for immunotherapy response. By quantifying the expression levels of these genes, clinicians can identify an immunosuppressive microenvironment enriched in myeloid-derived suppressor cells (MDSCs) and regulatory T cells (Tregs), thereby enabling improved patient stratification and the early identification of individuals who are likely to exhibit resistance to conventional 5-FU-based therapies [57].

The identification of senescent cells in vivo is significantly enhanced by the SenMayo gene set, a high-fidelity panel of 125 genes designed to overcome the limitations of traditional single-marker detection in single-cell transcriptomics. Developed through a rigorous cross-species analysis, this signature consists primarily of 83 SASP factors (such as IL1A, CXCL8, and CCL2) along with transmembrane and intracellular proteins, providing a robust framework for monitoring senescent cell burden across diverse tissues including bone, brain, and adipose tissue. Clinical and preclinical validations demonstrate that SenMayo successfully tracks the genetic and pharmacological clearance of senescent cells, better than other existing signatures that predict biological ageing. Notably, when applied to scRNA-seq datasets, the signature identified monocytic populations (CD14^+^/CD16^+^) and mesenchymal cells as central regulators of senesce, revealing the MIF (macrophage migration inhibitory factor) and platelet and endothelial cell adhesion molecule 1 (PECAM1) signalling pathways as critical mediators of immune evasion and microenvironmental remodelling. As a standardised and potentially universal signature, SenMayo serves as a foundational tool for the development of precision senotherapeutics, enabling accurate stratification of patients and objective assessment of treatment efficacy [58].

Closely related to cellular senescence, immunosenescence refers to the age-associated decline in the immune system function. Growing evidence suggests that immunosenescence contributes to cancer progression by accelerating tumour growth and promoting immune escape. Therefore, targeting immunosenescence has emerged as a promising therapeutic strategy to improve cancer prognosis; however, the lack of universally accepted biomarkers remains a major challenge. The immunosenescence risk model (ISRM), derived from a 90-gene immunosenescence gene set (ISGS), has been developed and validated across different cohorts. The ISRM utilises a five-gene signature (D40LG, IL7, CX3CR1, TLR3, and TLR2) to provide a robust framework for prognostic stratification in lung cancer. Patients categorised into the high-risk score showed worse survival and lower expression of immune checkpoints, contributing to resistance to immunotherapy. In contrast, the low-risk group shows a considerable benefit from immune checkpoint inhibitors. Beyond immunotherapy, the ISRM also serves as a predictor for targeted treatments, as high-risk patients demonstrate increased sensitivity to specific drugs like erlotinib. This model offers a critical tool for precision medicine, potentially identifying patients most likely to respond to different treatments [59].

Indeed, ageing is the primary risk factor for cancer, with approximately 60% of new malignancies occurring in individuals aged 65 years and older. High-resolution single-cell RNA sequencing (scRNA-seq) and the integration of large-scale single-cell datasets have enabled the identification of a conserved senescence signature characterised by *CDKN2A* upregulation across multiple tumour types, which is strongly associated with reduced overall survival and serves as an independent prognostic biomarker of tumour biological age and aggressiveness. To address the challenge of detecting rare senescent populations within the tumour microenvironment, advanced computational approaches based on artificial intelligence, deep learning, and multi-omics integration are increasingly being employed, improving detection sensitivity, enabling patient stratification, and facilitating the development of personalised interventions such as senolytic therapies [60].

The application of machine learning algorithms to large genomic datasets offers a powerful strategy for discovering clinically relevant genetic signatures also in rare and understudied tumours. Cholangiocarcinoma is a highly aggressive epithelial malignancy, with a poor prognosis and limited therapeutic options. Recent research utilising machine learning algorithms and TCGA datasets has led to the development of a novel cellular senescence-related signature (CSS) specifically for this cancer type. This signature encompasses a complex regulatory network of oncogenes and tumour suppressors, including *EZH2*, *CDK4*, *E2F1*, and *MDM2*, reflecting the dynamic interplay between proliferative drivers and cell cycle checkpoints during disease progression. Clinical evaluation has demonstrated that the CSS functions as a stable and powerful independent risk factor for predicting overall survival and immunotherapy response. Lower CSS scores are associated with reduced probability of immune escape and higher tumour mutation burden (TMB), suggesting that these patients may be more responsive to immune checkpoint inhibitors. In contrast, high CSS scores are associated with the enrichment of several oncogenic pathways, including glycolysis, p53 signalling, Hedgehog signalling, rapamycin complex 1 (mTORC1) signalling, DNA repair, hypoxia, Notch signalling, and angiogenesis, indicative of a more aggressive tumour phenotype. These findings are promising but are limited to transcriptomic data; therefore, further validation in prospective cohorts is necessary to confirm the robustness and applicability of the CSS model in real-world clinical settings [61].

All these models may provide valuable tools for patient stratification, and for investigating associations between molecular features, overall survival and potential clinical benefit from modern oncological therapies. Furthermore, the integration of artificial intelligence (AI) is opening new opportunities to move beyond static genomic analyses toward the investigation of how tumours may evolve over time. AI approaches, including deep learning such as graph neural networks, can integrate and analyse large-scale multi-omics with medical imaging and clinical data, potentially providing a more comprehensive characterisation of tumour heterogeneity within the same patient. Importantly, computational signatures derived from multi-omics data may identify molecular patterns associated with senescence but should not be considered sufficient evidence of functional senescence without complementary biological and functional validation. These computational tools may help identify patterns associated with tumour adaptation and potential resistance mechanisms during treatment; however, their ability to reliably predict such processes before they become clinically evident remains to be established. In the future, AI-driven “digital twins” and real-time decision support systems may contribute to more adaptive precision medicine, although their clinical implementation will require robust biological annotation, independent external validation and prospective evaluation [62]. Importantly, many current AI- and machine learning-based models remain retrospective and cohort-dependent. Overfitting, class imbalance, batch effects, incomplete biological annotation and differences between analytical platforms may limit their generalizability. Independent external validation, preferably in prospective and longitudinal cohorts, will therefore be essential before these approaches can be incorporated into clinical decision-making.

**Table 1:** Representative senescence-associated gene sets and molecular signatures relevant to cancer research.

Cancer/Tissue Type	Species	Analytical Construct	Gene Set/Model	Main Biological Insight	Datasets	Ref.
Cross-tissue (bone, brain, adipose)	Human and mouse	Senescence-associated gene set	SenMayo (125 genes)	Cross-tissue and cross-species identification of senescence-associated cells	GSE120446 GSE128423 GSE141595 GSE72815 GSE145265 GSE128770 GSE94832 GSE149590 GSE120221	[58]
Nasopharyngeal carcinoma	Human	Senescence-associated gene-set scoring	SenMayo (125 genes)	Senescence-associated subpopulations linked to tumour progression	GSE150430 GSE102349 GSE132112	[56]
Cholangiocarcinoma	Human	Gene-based risk model	CSS (6 genes: *EZH2*, *RNF2*, *CCNE1*, *E2F1*, *MDM2*, *CDK4*)	Senescence-associated risk stratification and treatment response	GSE145281 GSE89748 GSE107943 IMvigor210 GSE91061 TCGA-CHOL	[61]
Colorectal liver metastasis	Human	Machine learning-derived gene signature	5-gene signature: *CXCL1*, *SERPINE1*, *NDRG1*, *SRM*, *GATM*	Senescence-associated metabolic reprogramming and chemotherapy response	GSE213402 TCGA-COAD TCGA-READ Huashan Hospital cohort	[57]
Lung adenocarcinoma	Human and mouse	Gene-based risk model	ISRM (5 genes: *CD40LG*, *IL7*, *CX3CR1*, *TLR3*, *TLR2*)	Immunosenescence-associated immune microenvironment and treatment response	TCGA-LUAD GSE68465 GSE72094 GSE26939 GSE68571	[59]
Pancreatic ductal adenocarcinoma	Human	Gene-based risk model	3-gene prognostic signature (*CDK6*, *CENPA*, *MXD4*)	Senescence-associated prognosis and immune landscape	TCGA-PDAC, ICGC	[63]
Gastric cancer	Human	Gene-based risk model	Senescore (6 genes: *ADH1B*, *IL1A*, *SERPINE1*, *SPARC*, *EZH2*, *TNFAIP2*)	Senescence-associated subtypes and treatment response	TCGA-STAD GSE66229	[64]

## 5. Liquid Biopsy for Monitoring Therapy-Induced Senescence

Therapy-induced senescence and the accompanying SASP contribute to tumour plasticity, metastatic dissemination, and therapeutic resistance. Consequently, the longitudinal assessment of senescence- and EMT-associated biomarkers has emerged as a promising strategy to monitor tumour evolution, predict disease aggressiveness, and support therapeutic decision-making. While conventional tissue biopsies provide valuable molecular information, they are invasive and often unsuitable for repeated sampling. In contrast, liquid biopsy offers a minimally invasive approach for the dynamic assessment of tumour-derived biomarkers in body fluids such as blood, enabling real-time patient monitoring throughout disease progression and treatment.

Current liquid biopsy approaches encompass a broad spectrum of analytes, including soluble proteins and cytokines, circulating tumour cells (CTCs), circulating tumour DNA (ctDNA), circulating RNAs (ctRNAs), and extracellular vehicles (EVs) [65,66]. Multi-omic analyses of these circulating components, including proteomic, transcriptomic, and epigenomic profiling, have greatly expanded the discovery of biomarkers with diagnostic, prognostic, and predictive value across multiple cancer types [67].

The quantification of soluble proteins associated with SASP in plasma has become an increasingly common exploratory approach in clinical studies investigating systemic senescence burden and therapy-induced senescence in patients with cancer. Among the most extensively studied circulating candidates are GDF15, Activin A, IL-6, IL-8, SERPINE1, and several matrix metalloproteinases (such as MMP1), which have been associated with biological ageing, treatment-induced senescence, frailty, poor clinical outcome and therapeutic response in many contexts across multiple cancer types [5,68,69,70]. Nevertheless, the clinical utility of these soluble biomarkers remains limited by their lack of specificity, as most are also elevated in diverse inflammatory conditions, tissue injury and cancer itself.

These limitations have stimulated growing interest in EVs, which are membrane-enclosed particles naturally released by all cell types into the extracellular space [71]. As key mediators of intercellular communication, they transport a wide range of bioactive molecules, including proteins, lipids, DNA, mRNAs, and non-coding RNAs, to neighbouring and distant recipient cells. In contrast to soluble circulating factors, EVs protect their molecular cargo from degradation, especially for mRNA, while preserving membrane-associated proteins, ligands, and receptors that reflect the physiological and pathological status of the donor cell [72]. Their remarkable stability in biological fluids and accessibility from blood, urine, saliva, and other biofluids make them particularly attractive candidates for liquid biopsy.

Beyond their value as biomarkers, EVs actively participate in the regulation of multiple physiological and pathological processes, including cancer progression, cardiovascular diseases, neurodegeneration, metabolic disorders, and infectious diseases [73]. In the tumour microenvironment, tumour-derived EVs modulate immune responses, angiogenesis, extracellular matrix remodelling, angiogenesis, and even neural remodelling and metastatic dissemination through the transfer of proteins, lipids, and nucleic acids [74,75,76,77]. Their immunomodulatory activity has been associated with the transfer of molecules such as TGF-β, PD-L1, and ligands for the activating receptor Natural Killer Group 2 Member D (NKG2D), highlighting the broad biological relevance of EV-mediated communication [78,79,80,81,82,83].

**Figure 2:**
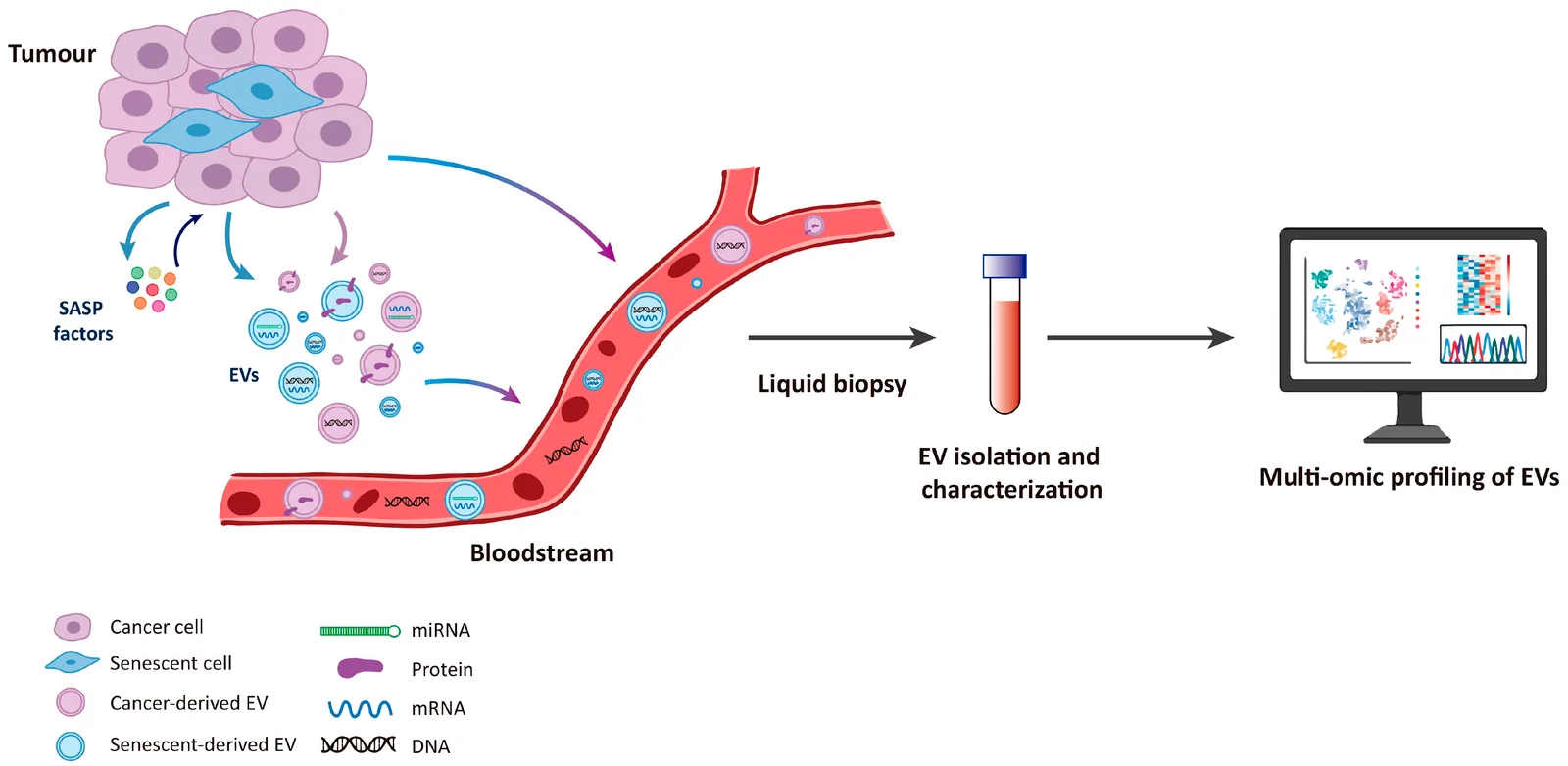
Role of extracellular vesicles (EVs) in liquid biopsy for cancer biomarker discovery. Cancer cells and senescent cancer cells release EVs and SASP factors into the tumour microenvironment. EVs carrying a heterogeneous cargo of miRNAs, mRNAs, proteins and DNA enter the bloodstream and can be isolated and/or characterised from liquid biopsy samples. Subsequent multi-omic profiling of EV cargo enables the identification of minimally invasive biomarkers with potential applications in cancer diagnosis, prognosis, patient stratification and treatment monitoring. In the context of senescence, different conditions or treatments could modify EV cargo biomarkers which could be detected in the profiling analysis. The colour of each arrow (blue or purple) corresponds to the colour of the cell from which the depicted mechanism originates.

Within the context of cellular senescence, EVs are recognised as an integral component of the SASP. Besides the secretion of soluble cytokines, chemokines, growth factors, and proteases, senescent cells exhibit a marked increase in EV biogenesis and release in response to DNA damage, oncogene activation, replicative exhaustion, and anticancer therapies. Importantly, senescent EVs carry a distinctive molecular signature that mirrors the senescent state of the donor cell and actively contributes to paracrine signalling. Their cargo has been shown to reinforce senescence in neighbouring cells, modulates inflammatory responses, remodels the tissue microenvironment, and may promote tumour progression, therapy resistance, and metastatic dissemination (Figure 2) [84,85].

Proteomic analyses have revealed an important remodelling of the protein composition of senescent EVs across different cell types and senescence-inducing stimuli, as compiled in the SASP Atlas database [68]. Several proteins have emerged as promising senescence-associated EV biomarkers. Among them, interferon-induced transmembrane protein 3 (IFITM3) is markedly increased in EVs generated during oncogene-induced senescence, where it functions as a key mediator of paracrine senescence through nuclear factor kappa B (NF-κB) activation in recipient cells [86]. Likewise, the Eph receptor A2 (EPHA2) tyrosine kinase is selectively enriched in EVs released by senescent cells and promotes cancer cell proliferation through ERK activation in recipient MCF-7 cells [87]. These proteins complement canonical EV markers, including the tetraspanins CD9, CD63, and CD81, together with accessory proteins such as ALG-2-interacting protein X (ALIX) and tumour susceptibility gene 101 protein (TSG101), which are commonly employed for EV characterisation [74,88].

The nucleic acid cargo of senescent EVs further expands their biological and biomarker potential. Multiple studies have demonstrated selective enrichment of senescence-associated microRNAs, including miR-433, miR-21-5p, miR-217, and miR-34a, which induce growth arrest and senescence in recipient cells through the modulation of targets such as Sirtuin 1 (SIRT1) or DNA (cytosine-5)-methyltransferase 1 (DNMT1) in recipient cells [89,90,91]. In addition, EVs transport mRNAs encoding canonical SASP components, including TGF-β and MMP-2, thereby extending the spectrum of functional signals transferred between cells [92]. Interestingly, the delivery of TGF-β mRNA, rather than only the mature protein, has been shown to induce paracrine senescence in recipient cells, illustrating the active contribution of EV-associated transcripts to the propagation of the senescent phenotype [93].

Another distinctive feature of senescent EVs is the presence of DNA fragments. EV-mediated DNA secretion constitutes an essential mechanism for maintaining cellular homeostasis by eliminating damaged or cytoplasmic DNA. Impairment of this process results in intracellular DNA accumulation, activation of the cyclic GMP–AMP synthase–stimulator of the interferon gene (cGAS-STING) pathway, permanent cell cycle arrest, and reinforcement of cellular senescence [94]. Otherwise, when released into the extracellular milieu, the DNA-containing EVs can also trigger DNA damage responses and senescence in recipient cells, providing an additional mechanism for propagation of senescence throughout the tissue.

The functional consequences of senescent EVs extend beyond the propagation of senescence itself. Increasing evidence indicates that these vesicles actively remodel the tumour microenvironment and influence tumour cell plasticity by promoting inflammatory signalling, extracellular matrix remodelling, immune evasion, and EMT, thereby facilitating metastatic dissemination [95]. Given the intimate relationship between senescence, SASP, and EMT, the molecular characterisation of senescent EVs represents an attractive opportunity beyond soluble proteins to monitor these dynamic processes during tumour progression.

From a translational perspective, EVs are particularly well suited for liquid biopsy because they are present in body fluids and preserve molecular information from their cells of origin with remarkable stability. The identification of senescence-specific EV signatures—including proteins, RNAs, and DNA—could therefore provide a non-invasive strategy to estimate the systemic senescent burden, monitor treatment-induced senescence, predict therapeutic response, and identify patients with increased risk of tumour progression or metastatic relapse (Figure 2). Importantly, the detection of a senescence-associated molecule in circulating EVs does not necessarily indicate an origin from therapy-induced senescent tumour cells, particularly in older patients or in the context of treatment-associated inflammation, stromal injury, or systemic senescence. Therefore, the specificity of EV-associated biomarkers for tumour cell senescence or therapy-induced senescence should be carefully assessed and validated in clinically relevant cohorts. On the other hand, methodological challenges such as pre-analytical variability, normalisation strategies, and assay reproducibility need to be addressed. Successful clinical implementation will ultimately require standardised, clinically scalable workflows and rigorous validation in large prospective patient cohorts.

Looking forward, the integration of conventional tissue biopsy and circulating markers such as EV-derived biomarkers with multi-omic profiling, longitudinal liquid biopsy analyses, and advanced computational approaches, including artificial intelligence and machine learning, is expected to substantially improve biomarker discovery and patient stratification [96,97,98,99]. Such integrative strategies will provide a more comprehensive view of the molecular interplay between senescence and EMT, ultimately facilitating precision oncology approaches based on dynamic, minimally invasive monitoring of tumour evolution.

## 6. Conclusions and Future Perspectives

The dynamic interplay between cellular senescence and epithelial-to-mesenchymal plasticity has emerged as a fundamental driver of tumour plasticity, contributing to cancer progression, metastatic dissemination, and therapeutic resistance. Beyond its role as a major functional feature of many senescent states, the SASP serves as a central paracrine mediator that promotes EMT, cancer stemness and microenvironmental remodelling. These findings highlight the dual role of therapy-induced senescence, which can initially suppress tumour growth but may ultimately promote tumour recurrence and therapeutic resistance if senescent cells persist. Consequently, senotherapeutic approaches, including senolytic and senomorphic agents, have emerged as potential components of one–two-punch therapeutic strategies aimed at eliminating or reprogramming persistent senescent cells, thereby disrupting these pro-tumourigenic interactions, overcoming therapy resistance. However, the clinical efficacy and safety of such approaches remain to be established, particularly given the potential effects of senolytic interventions on non-malignant tissues and the context-dependent effects of modulating SASP-associated signalling on immune surveillance.

At the same time, advances in multi-omics technologies, artificial intelligence, and liquid biopsy are transforming our ability to characterise and monitor these dynamic processes. The integration of multimodal senescence-related signatures with machine learning algorithms may facilitate patient stratification and the investigation of molecular patterns associated with tumour progression, while liquid biopsy biomarkers may provide minimally invasive approaches for monitoring tumour evolution, although their clinical utility remains to be established. Together, these advances open new avenues for deciphering tumour plasticity and translating this knowledge into precision oncology strategies through more precise biomarker-guided and personalised therapeutic strategies.

Future studies should prioritise longitudinal analyses to define the temporal evolution of TIS, mechanistic approaches to clarify its causal relationships with SASP, EMT and therapeutic resistance, and multimodal strategies to distinguish senescence from transient stress or other non-proliferative states. Clinically, senescence-associated biomarkers and senotherapeutic strategies will require prospective validation, appropriate patient selection, optimal treatment timing, and careful assessment of efficacy and toxicity before their integration into clinical practice.

## Data Availability

No new data were created or analysed in this study. Data sharing is not applicable to this article.

## Abbreviations

The following abbreviations are used in this manuscript:

TIS	Therapy-induced senescence
SASP	Senescence-associated secretory phenotype
EMT	Epithelial-to-mesenchymal transition
CSC	Cancer stem cell
MET	Mesenchymal-to-epithelial transition
SAS	Senescent-associated secretome
ECM	Extracellular matrix
ROS	Reactive species oxygen
BTZ	Bortezomib
TMZ	Temozolomide
LUAD	Lung adenocarcinoma
NSCLC	Non-small-cell lung cancer
BLCA	Bladder urothelial carcinoma
OXPHOS	Oxidative phosphorylation
AI	Artificial intelligence
ISRM	Immunosenescence risk model
ISGS	Immunosenescence gene set
CCS	Cellular senescence-related signature
CTC	Circulating tumour cell
EV	Extracellular vesicle
